# When You Can’t Play Sports: The Impact of the COVID-19 Pandemic on Motivational and Emotional Experiences in Coach-Athlete Dyads

**DOI:** 10.3390/ijerph192113944

**Published:** 2022-10-27

**Authors:** Marieke Fonteyn, Tom Loeys

**Affiliations:** Department of Data-Analysis, Faculty of Psychology and Educational Sciences, Ghent University, B-9000 Ghent, Belgium

**Keywords:** COVID-19 pandemic, coach–athlete relationship, passion for sport, basic psychological needs, affective experiences in sport, Actor–Partner Interdependence Model

## Abstract

(1) Background: This study aimed to assess the influence of the COVID-19 pandemic on athletes’ and coaches’ experiences. Following the Dualistic Model of Passion and the Self-determination Theory, the objectives of this study were to investigate whether the COVID-19 pandemic and its restrictions affected athletes’ and coaches’ passion experiences, emotional experiences and basic psychological needs while engaging in their sport activities. Furthermore, the relationship between passion and emotional experiences as well as between passion and the basic psychological needs were explored; (2) Methods: 87 coach-athlete dyads, active at the recreational or competitive level in an individual sport, participated in the study. Using a cross-sectional dyadic design, athletes and coaches reported separately on their passion experience, emotional experiences and basic psychological needs in the previous two weeks; (3) Results: In total, 30 dyads were impacted by the COVID-19 pandemic, while 57 were not. Athletes’ obsessive passion as well coaches’ negative affect were larger in impacted dyads, while athletes’ positive affect was lower in that group compared to the not-impacted group. Moderated Actor–Partner Interdependence Models revealed that coaches’ obsessive passion was more strongly related to their negative affect in coach–athlete dyads that were not impacted by the COVID-19 pandemic than in dyads that were impacted. Furthermore, the harmonious passion of coaches was more strongly associated with athletes’ need satisfaction and need frustration in impacted dyads, while also the athletes’ harmonious passion in impacted dyads was more strongly associated with coaches’ need satisfaction; (4) Conclusions: Less positive outcomes and more negative outcomes were observed in both athletes and coaches that were impacted by the COVID-19 pandemic. On the other hand, the COVID-19 pandemic may have suppressed the negative effects of coaches’ obsessive passion on their negative affect, but strengthened the positive impact of coaches’ harmonious passion on the athletes’ need satisfaction and vice versa.

## 1. Introduction

In spring 2020, the COVID-19 pandemic spread worldwide [1]. The pandemic not only affected the lives of almost the entire humanity, but governments were also forced to implement severe measures to curb the spread of the virus. As in other countries, the restrictions in Belgium included physical distancing from others and the recommendation to make only essential journeys. This also entailed a harsh impact on sports activities. Competitions and sport events were frequently postponed or suspended, and athletes and coaches were often prevented from exercising and coaching [2]. All of a sudden, the goals and prospects these athletes and coaches had in mind were knotted. Clearly, the pandemic induced a lot of uncertainty and doubt, which may have affected the motivational and emotional experiences of both athletes and coaches [3,4,5]. The aim of this study was therefore to investigate the impact of the COVID-19 pandemic on passion experiences, emotional experiences and the basic psychological needs of athletes and coaches from a dyadic perspective [6].

### 1.1. Passion for Sport

Due to the COVID-19 pandemic and the imposed restrictions athletes and coaches were challenged in the way they engaged in the activity they are passionate about: sports. The Dualistic Model of Passion (DMP) [7,8] defines passion as “a strong inclination toward a specific object, activity, concept or person that one loves (or at least strongly likes), highly values, invests time and energy in on a regular basis, and that is part of one’s identity” [7] (p. 33). For many athletes and coaches, engaging in sports meets these criteria [8]. However, athletes’ and coaches’ investments in terms of time and energy were often compromised by the COVID-19 pandemic, and so their passion experiences may have been pressurized. In the next paragraph we introduce the two types of passion that are distinguished by the DMP and elaborate on the consequences of these two types of passion. We then focus on how the COVID-19 pandemic might have affected athletes’ and coaches’ passion and emotional experiences as well as their basic psychological needs.

### 1.2. HP and OP and Their Consequences

The Dualistic Model of Passion differentiates between two types of passion. The distinction between these two types is based on how the passionate activity is ingrained in someone’s identity. Harmonious passion (HP) can be seen as an autonomous and more active type of passion. Here, an individual willingly accepts the activity as important and freely engages in the activity. A harmoniously passionate activity is essential, but not overwhelming for an individual and so, the activity is in harmony with other parts of one’s identity and other activities in one’s life. Obsessive passion (OP), by contrast, can be seen as a more controlled and passive type of passion in which an individual seems to become a slave to the passionate activity. The individual experiences internal or external pressure to engage in the activity. As the individual cannot help but to engage in the activity; obsessively passionate people often experience a lot of difficulties in balancing the different domains of life [7,8,9].

This tension between HP and OP also extends to the affective and motivational experiences with which both types of passion are related. Generally, the DMP expects HP to be associated with more adaptive outcomes, while OP is assumed to be less contributive to such desirable outcomes and sometimes even leads to maladaptive ones [7,8,9]. When it comes to the association between passion and affect, studies in sport and exercise mainly support the predictions of the DMP [7,9]. Research focused on athletes [8,10,11], coaches [12] and referees [13]. In almost all studies a positive association was found between harmonious passion and the experiences of positive affect or positive emotions, whereas obsessive passion was positively related to the experiences of negative affect and negative emotions [8,10,11,12,13]. Additionally, some studies found a negative association between harmonious passion and negative affect [11], while others did not find an association between obsessive passion and positive affect on the one hand and harmonious passion and negative affect on the other hand [10,13].

Passion for sport, however, is not only associated with positive or negative affect. Several studies also explored the association between passion and the basic psychological needs [14,15,16,17]. The Self-determination Theory (SDT) discerns three basic psychological needs that are essential for individuals to thrive [18,19]. The need for autonomy is a desire to be able to make free and volitional decisions. This need represents the sense of personal initiative regarding one’s own choices [19,20]. The need for competence embodies the desire to master one’s environment or interact effectively with it [19,21]. Finally, the need for relatedness represents the desire to have meaningful and close connections and relationships with significant others and to feel belongingness [19,22]. Further, SDT posits that an individual proactively searches for and engages in activities that are fulfilling one’s basic psychological needs in order to stimulate personal growth [18,19,23]. In nature, passionate activities have the potential to serve as one such activity [7,18]. For a passionate swimmer, swimming, for example, will provide feelings of autonomy—when the engagement is free and volitional—as it reflects a part of one’s identity. Additionally, when this swimmer engages in swimming on a regular basis with other athletes, the swimmer will not only become more skilled and competent—which stimulates the need for competence—but will also have the opportunity to develop relationships and friendships with other athletes—which stimulates the need for belongingness. As such, it is mainly HP that may lead to need satisfaction, whereas the feelings of internal or external pressure, characteristic for OP, may hinder or diminish those experiences of need satisfaction.

Research in sport and exercise on the association between passion and need satisfaction predominantly focused on athletes only and indicates that when engagement in sport is driven by HP, relative to OP, individuals generally experience more sense of autonomy, relatedness and competence. Obsessively passionate individuals, however, may experience some kind of need satisfaction when engaging in the passionate activity, but the compulsive nature of this type of passion keeps them from experiencing equal levels of need satisfaction while engaging in their sport [16,24,25].

To the best of our knowledge, studies focusing on the relationship between passion and need frustration in sport on the other hand are relatively sparse. Need frustration can be seen as the flip side of need satisfaction and embodies the thwarting of someone’s needs. Indeed, while participation in sport may unfold psychological growth by feeding one’s needs when mastering new skills and techniques (competence satisfaction), when feeling freedom of choice (autonomy support), or when experiencing warm and close relationships with team members or coaches (relatedness satisfaction), the same sport setting can be extremely pressuring and demanding. Sport can equally undermine psychological and physical growth by feelings of being controlled by the coach or teammates (autonomy frustration), feelings of incompetence (competence frustration) or rejection and exclusion from the team (relatedness frustration) [26]. Actively undermining one’s needs can, on its turn, lead to an impaired psychological growth and even malfunctioning, pathogenic outcomes, psychopathology and ill-being [26,27]. Need frustration is different from need deprivation, which represents an absence or a lack of need satisfaction [27].

### 1.3. The Impact of COVID-19 and the Present Study

The COVID-19 pandemic affected coaches and athletes at various levels [2,3,4,28]. The aims of our study were two-fold. First, we wanted to explore how the COVID-19 pandemic affected passion experiences, emotional experiences and basic psychological needs in athletes and coaches (e.g., by being prevented from exercising or coaching, by quarantine restriction) (Hypothesis 1). Second, we wanted to investigate the association between the two types of passion and affective experiences on the one hand and between the two types of passion and the psychological needs on the other hand, and the impact of the COVID-19 pandemic on those associations (Hypothesis 2). 

We hypothesized that, both in athletes and coaches, the harmonious passion experiences (Hypothesis 1a), positive affective experiences (Hypothesis 1b) and need satisfaction experiences (Hypothesis 1c) were lower in coach–athlete dyads that were reported as being affected by the COVID-19 pandemic. For obsessive passion (Hypothesis 1d), negative affect (Hypothesis 1e) and need frustration (Hypothesis 1f), we expected that dyads that were affected by the pandemic reported higher levels on those outcomes compared to the dyads that weren’t affected.

Second, we expected both in athletes and coaches a positive relationship between harmonious passion and positive affect (Hypothesis 2a) and between obsessive passion and negative affect (Hypothesis 2b). Concerning the association between harmonious passion and the basic psychological needs, we hypothesized a positive association between HP and need satisfaction (Hypothesis 2c) and a negative association between HP and need frustration (Hypothesis 2d). Finally, for the association between obsessive passion and the psychological needs, we expected the relationship between OP and need satisfaction (Hypothesis 2e) and the association between OP and need frustration (Hypothesis 2f) to be positive. Further, we explored the impact of the COVID-19 pandemic on each of those associations. As those moderation effects are considered as exploratory, we did not formulate specific hypotheses about them.

This study extends the previous literature in an important way due to the dyadic perspective that is taken. Scholars recently showed the importance of considering both athletes’ and coaches’ perspectives while examining coach–athlete dynamics [6]. Indeed, coaches and athletes depend on one another and influence each other in their collaboration [29,30]. By investigating the impact of the pandemic on passion, emotional experiences and the basic psychological needs from a dyadic perspective, new and more fine-grained insights may arise. In this study we therefore use the Actor–Partner Interdependence Model in which both actor and partner effects for athletes and coaches are considered [31,32,33]. Actor effects can be seen as intrapersonal effects and represent the effect of an athlete’s (coach’s) predictor variable on the athlete’s (coach’s) own outcome variable. Partner effects can be seen as interpersonal effects and represent the effect of an athlete’s (coach’s) predictor variable on a coach’s (athlete’s) outcome variable. The use of a dyadic perspective is relatively sparse in sport psychology while it is important to simultaneously consider actor and partner effects [6]. In our Hypotheses 2a through 2f, we therefore implicitly assumed the four APIM effect to be present. That is, we hypothesized the presence of the actor effect in coaches, the actor effect in athletes, the partner effect from athletes to coaches and the partner effect from coaches to athletes for all six associations under consideration.

## 2. Materials and Methods

### 2.1. Participants

In total, 87 coach–athlete dyads that were active at the recreational or more competitive level in individual sports, participated in the study (Table 1). Dyads for which the athlete or the coach did not fill in the questionnaire and/or did not agree with the informed consent (i.e., 17 dyads in total) were excluded from the study. In the sample, there were more male (67.82%) than female coaches (32.18%), but there were more female (62.07%) than male athletes (37.93%). The athletes (M_age_ = 28.63, SD = 12.95) were active in different individual sport disciplines (e.g., tennis, track and field, gymnastics) with on average almost 11 years of experience in their sport (SD = 8.67). They performed at different competition levels: most of them participated on the recreational or amateur level (32.18%), followed by the competitive level (29.89%), the high-competitive level (26.44%) and the low-competitive level (11.49%). The coaches (M_age_ = 40.86, SD = 14.65) reported on average almost 14 years of coaching experience (SD = 11.03) and more than 14 h of coaching per week (SD = 12.85). Most of them (88.51%) reported having some type of coaching degree. The mean length of the coach–athlete relationship was 4.42 years (SD = 3.88). Athletes reported spending on average 5.13 h of training per week with their coach (SD = 3.94), of which 1.59 h (SD = 2.61) were in one-to-one contact with their coach.

### 2.2. Procedure

Coach–athlete dyads were recruited from November 2021 until March 2022. During these months, different measures were imposed. In November, December and January there was, amongst others, a requirement to wear a mouth mask in indoor sport centers, the need for a Covid Safe Ticket in many indoor sport facilities and it was forbidden to have audiences at indoor competitions. Starting from February, many of these restrictions were cancelled. During the entire recruitment period, social distancing was mandatory or recommended, whereas training was forbidden or competitions were suspended when people were infected by the virus [34,35]. Participants were recruited via online advertisement on social media (e.g., LinkedIn, Facebook, Instagram), personal contacts of the involved researchers and posters that were distributed on relevant locations (e.g., sport clubs). Master students helped in the process of data collection. Only athletes whose coach agreed to participate (or vice versa, only coaches whose athlete agreed to participate) were allowed to participate. Thereafter, each participant received more information about the study, an informed consent and a unique survey web-link that guided the participants to an online survey. Both for coaches and for athletes the online questionnaire took about 30 min to complete. Participants who didn’t fill in the questionnaire within a week after the first invitation received a reminder to complete the questionnaire. Each participant received up to five reminder e-mails. The research was conducted in accordance with the ethical guidelines of the General and Specific Ethical Protocol of the Faculty of Psychology and Educational Sciences of Ghent University.

### 2.3. Measures

#### 2.3.1. Background Variables

Athletes’ age, years of experience, competition level and gender were assessed, as well as coaches’ age, years of experiences, hours of coaching per week and gender. Furthermore, athletes also reported on the years of collaboration with their coach and the hours of contact they had with their coach per week. Finally, both athletes and coaches had to report on the impact of the COVID-19 pandemic on their sport participation by answering the question whether they were, at the moment of participation, affected by the pandemic or the restrictions that were in force at that time to curb the spread of the virus. Dyads of which both dyad members indicated that their sport participation was impacted by the pandemic were seen as being affected by the pandemic (N_impacted_ = 30). All other dyads were treated as not-impacted dyads (N_not-impacted_ = 57).

#### 2.3.2. Passion for Sport and Coaching

Passion for sport and coaching was measured in athletes and coaches, respectively, by means of the Passion Scale [8]. The backtranslation procedure was used to translate the original instrument into Dutch. The original English instruments were first translated into Dutch by a researcher fluent in Dutch. The back translations were done by an independent third person (i.e., a person with a master’s degree in languages). A third person, who was fluent in English, inspected and compared the original and back-translated items on their equivalence. Non-equivalent translations were discussed by the researchers to arrive at consensus. The Passion Scale consists of two six-items scales assessing harmonious (e.g., “Doing sport is in harmony with other things that are part of me”) and obsessive passion (e.g., “I have almost an obsessive feeling for coaching”). All items were measured on a seven-point Likert scale, ranging from 1 (“I do not agree at all”) to 7 (“I strongly agree”) and were formulated with reference to the preceding two weeks. For both scales a composition score was calculated by taking the mean for coaches and athletes. The Passion Scale in the adapted version for athletes and coaches has shown high levels of validity and reliability in previous studies [8,10,12]. The internal consistencies in this study were α_harmonious passion_ = 0.64 and α_obsessive passion_ = 0.73 for athletes and α_harmonious passion_ = 0.71 and α_obsessive passion_ = 0.78 for coaches.

#### 2.3.3. Basic Psychological Needs

Basic psychological need satisfaction and frustration were measured in both athletes and coaches through an adapted version of the Basic Psychological Need Satisfaction and Frustration Scale (BPNSFS) [36]. The sport-specific versions of the questionnaire for athletes and coaches [37,38] were adapted to a more dyadic perspective in which the coach–athlete relationship was more explicitly targeted. The items were formulated with reference to the preceding two weeks. For both need satisfaction and need frustration two items were used per need, resulting in six items for need satisfaction (e.g., “While practicing sports, I felt connected with my coach”) and six items for need frustration (e.g., “I feel I have no other choice but to coach athletes’). All 12 items were answered on a seven-point Likert scale ranging from 1 (“Totally not agree”) to 7 (“Totally agree”). Composite scores for need satisfaction and need frustration were obtained for both athletes and coaches by calculating the mean. The internal consistencies for athletes in the study were α_satisfaction_ = 0.78 and α_frustration_ = 0.61. The internal consistencies for coaches were α_satisfaction_ = 0.77 and α_frustration_ = 0.78.

#### 2.3.4. Positive and Negative Affect

The affective experiences while coaching or participating in sport of coaches and athletes, respectively, were measured via the Positive and Negative Affect Schedule (PANAS) [39]. A Dutch translation of the original scale was made and validated in prior research [40]. The PANAS includes two scales which distinguish positive affect (PA) and negative affect (NA). For this study, a selection of only ten items that were applicable to and relevant for sports was made. Each of the items reflect emotional experiences. Five of them were used to assess PA (e.g., interested) and five were used to assess NA (e.g., irritable). Athletes and coaches were asked to evaluate each of the emotions while thinking on doing sport and coaching, respectively. The items were evaluated on a five-point Likert scale ranging from 1 (“Very slightly or not at all”) to 5 (“Extremely”) and all items were formulated with a reference to the preceding two weeks. For both scales a sum-score was calculated for athletes and coaches. The internal consistencies for coaches were α_positive affect_ = 0.82 and α_negative affect_ = 0.69. The internal consistencies for athletes were α_positive affect_ = 0.84 and α_negative affect_ = 0.83.

### 2.4. Plan of Analysis

In a first step we compared the background variables between impacted and not-impacted dyads. For continuous background variables, normality was assessed first, and two sample Welsh t-tests were performed thereafter. For categorical background variables a χ^2^-test was used. All variables that were significantly different between both groups were included in all further analyses as potential confounders. To test Hypotheses 1a through 1f, we first assessed the normality of the outcomes. Thereafter, we fitted a multivariate linear regression model for each outcome in coaches and athletes simultaneously, with groups as a predictor while adjusting for the identified confounders. To test Hypotheses 2a through 2f, the Actor–Partner Interdependence Model (APIM) was fitted using structural equation modeling via the R-package lavaan [41]. In those APIMs we allowed for different actor and partner effects in both groups and adjusted for confounders. All analyses were performed at the 5% significance level. The study was originally designed to have 80% power to detect moderate actor and partner effects in the overall sample.

## 3. Results

### 3.1. Preliminary Analysis

Baseline demographic information can be found in Table 1. The mean number of hours of coaching per week (*t*(85) = 2.640, *p* = 0.010) and the total hours of contact per week (*t*(46) = −2.279, *p* = 0.027) were significantly different between the dyads that were and were not affected by the COVID-19 pandemic. For the competition level, a significant difference between both groups (*χ^2^*(3) = 8.8, *p* = 0.003) was found as well. However, after controlling for the hours of contact per week and the total hours of coaching per week, this difference was no longer significant. To avoid multicollinearity issues, only the hours of contact per week and the total hours of coaching per week variables were therefore included as background variables in all further models. Descriptive statistics and bivariate correlations among the study variables can be found in Table 2 for athletes and Table 3 for coaches.

### 3.2. Differences in Passion, Needs and Affect between Impacted and Not-Impacted Dyads

Table 2 and Table 3 present the raw means by group for all study variables. Both in athletes and coaches, higher means were observed for the adaptive outcomes in the group that was not impacted, while lower means were observed for the non-adaptive outcomes in that group. To avoid erroneous conclusions due to potential confounding we did not compare the raw means directly but used regression to address Hypotheses 1a through 1f. After adjusting for contact hours and hours of coaching, significant mean level differences between impacted and not-impacted dyads were found for athletes’ obsessive passion (*z* = 3.115, *p* = 0.002) and positive affective experiences (*z* = −2.003, *p* = 0.045) and for coaches’ negative affective experiences (*z* = 2.119 *p* = 0.034). Further, the mean level differences between the impacted and not-impacted dyads for coaches’ need satisfaction (*z* = −1.939, *p* = 0.053) and positive affective experiences (*z* = −1.648, *p* = 0.099) were marginally significant.

### 3.3. Moderated Actor-Partner Interdependence Models

**Figure 1:**
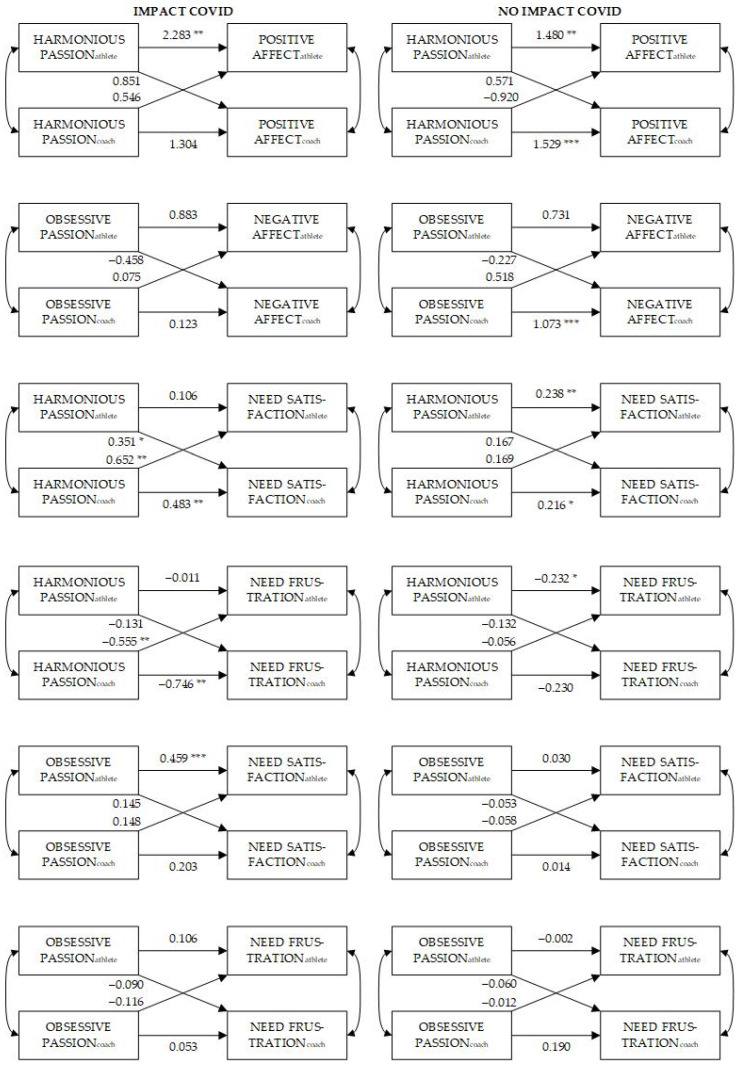
A visual representation of the actor and partner effects of the six moderated Actor–Partner Interdependence models that were fitted. In the left panel, the results of the dyads that were impacted by the pandemic can be found, whereas the results of the not-impacted dyads are displayed in the right panel. *** *p* < 0.001; ** *p* < 0.01; * *p* < 0.05.

The estimated actor and partner effects for all six associations under study are presented in Figure 1 for the impacted and not-impacted groups separately. For clarity, we omitted the effects of the number of hours of coaching per week and the total hours of contact per week from the path diagrams. Below, we discuss each of the six associations.

#### 3.3.1. Harmonious Passion and Positive Affect

Evidence is found for the athletes’ actor effect in both impacted (*z* = 2.954, *p* = 0.003) and not-impacted dyads (*z* = 3.481, *p* = 0.001), while only the coaches’ actor effect was found for not-impacted dyads (*z* = 4.192, *p* < 0.001). In all those cases, one’s own harmonious passion was positively associated with one’s own positive affect. No evidence was found for partner effects. Further, none of the actor effects were significantly different between both groups.

#### 3.3.2. Obsessive Passion and Negative Affect

Only the coaches’ actor effect was significantly positive for not-impacted dyads (*z* = 3.919, *p* < 0.001). That is, when the dyad was not impacted by COVID-19, obsessive passion in the coaches was positively associated with their negative affect. Furthermore, there was a significant difference between impacted and not-impacted dyads for this actor effect in coaches (*z* = 2.106, *p* = 0.035).

#### 3.3.3. Harmonious Passion and Need Satisfaction

The athletes’ actor effect in not-impacted dyads (*z* = 2.671, *p* = 0.008), the two coaches’ actor effects (*z* = 2.592, *p* = 0.010 for impacted dyads and *z* = 2.148, *p* = 0.032 for not-impacted dyads), the partner effect from coaches to athletes in impacted dyads (*z* = 3.398, *p* = 0.001) and the partner effect from athletes to coaches in impacted dyads (*z* = 2.244, *p* = 0.025) were all found to be significantly positive. A significant difference between impacted and not-impacted dyads was found for the partner effect from coaches to athletes (*z* = −2.224, *p* = 0.026). Here, both effects were positive, however the association between coaches’ harmonious passion and athletes’ need satisfaction was more pronounced in impacted than in not-impacted dyads.

#### 3.3.4. Harmonious Passion and Need Frustration

The athletes’ actor effect in not-impacted dyads (*z* = −2.519, *p* = 0.012), the coaches’ actor effect in impacted dyads (*z* = −3.068, *p* = 0.002) and the partner effect from coaches to athletes in impacted dyads (*z* = −2.805, *p* = 0.005) were statistically significant. All associations were in the hypothesized direction. The partner effect from coaches to athletes was found to be significantly different between impacted and not-impacted dyads (*z* = 2.229, *p* = 0.026). Here, the negative association between the coaches’ harmonious passion and the athletes’ need frustration was more pronounced for impacted dyads than it is for not-impacted dyads.

#### 3.3.5. Obsessive Passion and Need Satisfaction

Only the actor effect for athletes from impacted dyads was significantly positive (*z* = 3.895, *p* < 0.001). In athletes that were impacted by COVID-19, more obsessive passion was associated with higher need satisfaction. This effect was significantly different between impacted and not-impacted dyads (*z* = −3.113, *p* = 0.002).

#### 3.3.6. Obsessive Passion and Need Frustration

No significant associations were found between obsessive passion and need frustration in either group for coaches and athletes.

## 4. Discussion

The COVID-19 pandemic hit both athletes and coaches right in their heart. They were challenged in the way they engaged in their sport activities and had to respond with flexibility to this situation [3,4,5]. The present study aimed to investigate the impact of this demanding and unfamiliar situation on the motivational and emotional experiences, not only for athletes in individual sports, but also for their coaches. Previous studies that investigated passion, emotional experiences and the basic psychological needs predominantly focused on athletes only [8,10,11,24,25]. By further relating the experiences of athletes to the experiences of coaches (and vice versa), we shed a new and unique light on their bidirectional influences in times of doubt and uncertainty. Indeed, a bidirectional contamination between the coaches’ and athletes’ experiences may be present. More specifically, coaches may, for example, transfer their passion experiences to athletes’ affective and motivational experiences, and vice versa. However, to this day, little attention has been given to this potential bidirectional influence, let alone how it is impacted by COVID-19.

### 4.1. The Impact of COVID-19 on Motivational and Emotional Experiences

The first aim of this study was to explore whether there were differences in passion experiences, need satisfaction and frustration and general affective experiences between coach–athlete dyads that were impacted by the pandemic and coach-athlete dyads that were not (Hypotheses 1a to 1f). Athletes’ obsessive passion was on average higher in the impacted coach–athlete dyads than in the not-impacted dyads, whereas a reserved pattern was found for athletes’ positive affect. Coaches from impacted dyads experienced on average more negative affect than coaches from not-impacted dyads. These findings might be relevant and important to consider in sport practices. Apart from the pandemic, other challenging situations may occur, which on its turn may impact the motivational or emotional experiences of both coaches and athletes. Monitoring those experiences and trying to mitigate their negative consequences may improve both athletes’ and coaches’ future sport experiences. In addition, following the Self-determination theory, studies showed that the need-based experiences of coaches and athletes depend, on a macro-level, on governmental and sport federation policies [42]. Therefore, it is of the upmost importance to be attentive for nurturing the needs of autonomy, relatedness and competence at all times, especially in challenging situations such as the COVID-19 pandemic when considering restrictions.

### 4.2. Passion and Its Consequences on Affective Experiences and Psychological Needs

The second aim of the present study was to investigate the association between passion and affective experiences and psychological needs (Hypotheses 2a to 2f), and to explore the potential impact of COVID-19. First, we inspected the association between harmonious passion and affect. In line with prior research [8,10,11,13], we found that athletes’ and coaches’ harmonious passion was positively related to their own positive affective experiences, and their obsessive passion positively related to their own negative affective experiences. This emphasizes the role HP and OP play for both athletes’ and coaches’ affective experiences. The only significant difference in actor effects between the impacted and not-impacted group was a stronger association between coaches’ obsessive passion and their negative affect in the absence of COVID-19 impact. In contrast to our expectations, no evidence for partner effects was found in the association between passion and affective experiences.

Interestingly, evidence for such partner effects was found for the relationship between harmonious passion and psychological needs. The coaches’ harmonious passion was positively associated with the athletes’ need satisfaction and negatively with the athletes’ need frustration in the group that was impacted by COVID-19. As such, when dyads were influenced by the pandemic, coaches’ harmonious passion could have served as a resource for need satisfaction in athletes and as a protective factor against experiences of need frustration in athletes. In addition to the partner effect from coaches to athletes, the partner effect from athletes’ harmonious passion to coaches’ need satisfaction was found in impacted dyads too. The importance of these effects is not to be underestimated, as it indicates that a partner’s passion experiences may stimulate one’s own need satisfaction, especially when the situation is challenging. In addition, both effects together provide evidence for the bidirectional nature of the coach–athlete relationship [6] and indicate that, in uncertain times like the COVID-19 pandemic, athletes’ harmonious passion contributes to coaches’ need satisfaction and vice versa. We recognize however that these moderated effects should cautiously be considered since those effects were rather exploratory in nature in this study. The results, therefore, need further replication in other types of challenging situations in sports.

Somewhat surprisingly, the association between obsessive passion and need satisfaction and between obsessive passion and need frustration did not show any significant effects but one. Only the athletes’ obsessive passion was found to be positively associated with their own need satisfaction in impacted dyads. Possibly, and in line with prior research [43], the passionate activity is one of the few activities from which need satisfaction can be obtained for obsessive passionate people, even when the situation is challenging. In contrast, for harmoniously passionate people several activities provide feelings of need fulfillment.

### 4.3. Limitations and Future Directions

A first limitation of the present study was the cross-sectional design. Such a design does not allow drawing causal conclusions, nor does it consider the longitudinal nature of the impact of COVID-19. A longitudinal design would not only provide additional support on the sequence of effects between passion and its outcomes, but also will tell us more on the stability of the effects over time and the existence of cross-lagged effects in coach-athlete dyads.

Furthermore, we need to be careful to make causal statements on the impact of COVID-19 on motivational and emotional experiences. We found that mainly athletes at the competitive level were impacted by the pandemic. While we adjusted for the total hours of coach–athlete contact per week and the coaches’ hours of coaching per week to address potential confounding, there may still be other mechanisms at play. For example, is it the COVID-19 pandemic that stirred up athletes’ obsessive passion? Do the athletes that score on average higher on the obsessive passion measure report more often to be affected by the pandemic? A more objective measure of the impact of the COVID-19 pandemic rather than subjective reporting would have been desirable to address this question.

Third, this study only focused on individual sport athletes. However, the COVID-19 pandemic might have affected the dynamics in sport teams in different ways than in individual sports. Similar studies in team sports could provide an interesting extension of the present study. Furthermore, the COVID-19 measures caused many sports federations to suspend their activities. The data collection of this study took place in the transition period in which the restrictions were gradually dropped. As each of the federations had some autonomy in this process, some individual sports might not be present in the sample due to the measures that were still retained by a specific federation (e.g., making it impossible to practice, compete or coach).

Fourth, our study used self-reporting online questionnaires, which are considered a more subjective evaluation method. Future research could also include more objective measures of characteristics and behaviors of both coaches and athletes by an independent observer.

Finally, it is recommended for future studies to use a dyadic lens in the investigation of the dynamics in the coach–athlete relationship as well. Taken together, only limited evidence was found for the existence of the partner effects. Several explanations can be found for this lack of partner effects. First, it might be possible that coaches and athletes do not impact each other in (some of) the associations considered here. In such an actor-only oriented dyadic pattern, only one’s own passion affects one’s own affective experiences, without being impacted by one’s partner’s passion, for example. It might also be the case that only coaches and athletes that worked together for a longer period of time or with greater intensity strongly affect each other. Or perhaps the coaches’ or athletes’ personality traits may be determinant to one’s sensitivity of being influenced by one’s partner. Future research can shed some further light on these possible explanations. Furthermore, when investigating the coach–athlete relationship from a dyadic lens, the study sample should be adequately powered to the unique context. Since this study was originally designed to detect associations in the overall sample, the sample size of the present study may have been too small to detect medium actor and partner effects in each subgroup separately [44] and to detect differences between these two groups.

## 5. Conclusions

This study explicitly took a dyadic perspective in which the presence of bidirectional effects in the coach–athlete relationship was investigated [6]. The present study supported prior research by replicating many actor effects, but also revealed a few important partner effects. The study evidenced, for example, the importance of one’s partner harmonious passion experiences for the fulfillment of one’s own needs. This association turned out to be particularly important when being challenged by the COVID-19 pandemic. As many other situations in sport are equally challenging, the protective role coaches and athletes have towards each other may also apply in other situations. Future research should therefore focus on these bidirectional processes in sports in general and in other situations in which such uncertainty is present.

## Figures and Tables

**Table 1:** Demographic information for the total sample, the dyads that reported being affected by the pandemic and the dyads that reported not to be affected by the pandemic.

Baseline Characteristics	Total (*n* = 87)	Impact COVID (*n* = 30)	No Impact COVID (*n* = 57)
Athletes			
Age (years)			
Mean (SD)	28.63 (12.95)	25.20 (11.14)	30.44 (13.56)
Range	16–72	16–60	16–72
Competition level (*n*)			
High-competitive	23 (26.44%)	10 (33.33%)	13 (22.81%)
Competitive	26 (29.98%)	15 (50.00%)	11 (19.30%)
Low-competitive	10 (11.49%)	2 (6.67%)	8 (14.04%)
Recreational/amateur	28 (32.18%)	3 (1.00%)	25 (43.86%)
Sex (*n*)			
Male	33 (37.93%)	10 (33.33%)	23 (40.35%)
Female	54 (62.07%)	20 (66.67%)	34 (59.65%)
Experience (years)			
Mean (SD)	10.99 (8.67)	12.10 (8.63)	10.41 (8.71)
Range	0–45	3–45	0–44
Coaches			
Age (years)			
Mean (SD)	40.86 (14.65)	41.83 (15.47)	40.35 (14.31)
Range	21–78	21–66	21–78
Trainer degree (*n*)			
Yes	77 (88.50%)	28 (93.33%)	49 (85.96%)
No	10 (11.49%)	2 (6.67%)	8 (14.04%)
Sex (*n*)			
Male	59 (67.82%)	20 (66.67%)	39 (68.42%)
Female	28 (32.18%)	10 (33.33%)	18 (31.58%)
Experience in coaching (years)			
Mean (SD)	13.95 (11.03)	14.57 (10.55)	13.63 (11.35)
Range	0.5–42	2–42	0.5–36
Hours of coaching per week (hours)			
Mean (SD)	14.13 (12.85)	10.10 (7.04)	16.25 (14.65)
Range	2–60	3–30	2–60
Coach-athlete relationship			
Years of collaboration (years)			
Mean (SD)	4.42 (3.88)	4.93 (3.55)	4.16 (4.05)
Range	0.029–20	0.21–15	0.029–20
Total contact with coach (hours)			
Mean (SD)	5.13 (3.94)	6.55 (4.60)	4.39 (3.35)
Range	0–17.5	0–17.5	0–15
One-to-one contact with coach (hours)			
Mean (SD)	1.59 (2.61)	1.35 (2.12)	1.72 (2.85)
Range	0–15	0–6	0–15

**Table 2:** Descriptive statistics of and correlations between the athletes’ study variables.

	1	2	3	4	5	6
1. Harmonious passion	-	0.065	0.142	−0.053	0.476 **	−0.031
2. Obsessive passion	−0.052	-	0.579 ***	0.124	0.138	0.220
3. Need satisfaction	0.339 **	0.048	-	−0.416 *	0.088	0.111
4. Need frustration	−0.369 **	−0.002	−0.521 ***	-	0.104	0.208
5. Positive affect	0.410 **	0.041	0.013	−0.089	-	−0.039
6. Negative affect	−0.476 ***	0.224	−0.196	0.285 *	−0.523 ***	-
Impacted dyads (*n* = 30)						
M_athletes_	5.306	3.850	5.750	1.933	19.067	9.600
SD_athletes_	0.675	0.942	0.747	0.830	3.258	3.607
Not-impacted dyads (*n* = 57)						
M_athletes_	5.272	3.289	6.018	1.673	19.368	8.947
SD_athletes_	0.872	1.166	0.616	0.547	3.155	4.232

Note: The correlations between the athletes’ variables for dyads that were affected by the pandemic are displayed above the diagonal, the correlations between the athletes’ variables for dyads that were not affected by the pandemic can be found below the diagonal. *** *p* < 0.001; ** *p* < 0.01; * *p* < 0.05.

**Table 3:** Descriptive statistics of and correlations between the coaches’ study variables.

	1	2	3	4	5	6
1. Harmonious passion	-	−0.077	0.406 *	−0.469 **	0.371 *	−0.146
2. Obsessive passion	−0.075	-	0.315	0.044	0.222	0.054
3. Need satisfaction	0.341 **	0.106	-	−0.357	0.590 ***	−0.127
4. Need frustration	−0.231	0.225	−0.561 ***	-	−0.241	0.318
5. Positive affect	0.498 ***	0.119	0.474 ***	−0.394 **	-	0.048
6. Negative affect	−0.003	0.519 ***	−0.137	0.331 *	−0.093	-
Impacted dyads (*n* = 30)						
M_coaches_	5.289	2.817	5.656	2.189	20.167	7.400
SD_coaches_	0.567	0.961	0.739	0.922	2.167	2.343
Not-impacted dyads (*n* = 57)						
M_coaches_	5.614	2.658	5.982	1.877	21.175	6.421
SD_coaches_	0.767	0.941	0.635	0.741	2.746	1.963

Note: The correlations between the coaches’ variables for dyads that were affected by the pandemic are displayed above the diagonal, the correlations between the coaches’ variables for dyads that were not affected by the pandemic can be found below the diagonal. *** *p* < 0.001; ** *p* < 0.01; * *p* < 0.05.

## Data Availability

Raw data and the scales that are used for this project will be made available by the first author upon request.

## References

[1] World Health Organization Coronavirus Disease (COVID-19) Pandemic. https://www.who.int/emergencies/diseases/novel-coronavirus-2019.

[2] Schinke R., Papaioannou A., Henriksen K., Si G., Zhang L., Haberl P. (2020). Sport Psychology Services to High Performance Athletes during COVID-19. Int. J. Sport Exerc. Psychol..

[3] di Fronso S., Costa S., Montesano C., di Gruttola F., Ciofi E.G., Morgilli L., Robazza C., Bertollo M. (2022). The Effects of COVID-19 Pandemic on Perceived Stress and Psychobiosocial States in Italian Athletes. Int. J. Sport Exerc. Psychol..

[4] Santi G., Quartiroli A., Costa S., di Fronso S., Montesano C., di Gruttola F., Ciofi E.G., Morgilli L., Bertollo M. (2021). The Impact of the COVID-19 Lockdown on Coaches’ Perception of Stress and Emotion Regulation Strategies. Front. Psychol..

[5] Costa S., Santi G., di Fronso S., Montesano C., di Gruttola F., Ciofi E.G., Morgilli L., Bertollo M. (2020). Athletes and Adversities: Athletic Identity and Emotional Regulation in Time of COVID-19. Sport Sci. Health.

[6] Fonteyn M., Haerens L., Vansteenkiste M., Loeys T. (2022). It Takes Two to Tango: Using the Actor-Partner Interdependence Model for Studying the Coach-Athlete Relationship. Psychol. Sport Exerc..

[7] Vallerand R.J. (2015). The Psychology of Passion: A Dualistic Model.

[8] Vallerand R.J., Blanchard C.M., Mageau G.A., Koestner R., Ratelle C.F., Leonard M., Gagné M., Marsolais J. (2003). Les Passion de l’ame: On Obsessive and Harmonious Passion. J. Pers. Soc. Psychol..

[9] Vallerand R.J., Verner-Filion J., Tenenbaum G., Eklund R. (2019). Theory and Research in Passion for Sport and Exercise. Handbook of Sport Psychology.

[10] Vallerand R.J., Rousseau F.L., Grouzet F.M.E., Dumais A., Grenier S., Blanchard C.M. (2006). Passion in Sport: A Look at the Determinants and Affective Experiences. J. Sport Exerc. Psychol..

[11] Philippe F.L., Vallerand R.J., Houlfort N., Lavigne G., Donahue E.G. (2010). Passion for an Activity and the Quality of Interpersonal Relationships: The Mediating Role of Emotions. J. Pers. Soc. Psychol..

[12] Lafrenière M.K., Jowett S., Vallerand R.J., Donahue E.G., Lorimer R. (2008). Passion in Sport: On the Quality of the Coach Athlete Relationship. J. Sport Exerc. Psychol..

[13] Philippe F.L., Vallerand R.J., Andrianarisoa J., Brunel P. (2009). Passion in Referees: Examining Their Affective and Cognitive Experiences in Sport Situations. J. Sport Exerc. Psychol..

[14] Curran T., Appleton P.R., Hill A.P., Hall H.K. (2013). The Mediating Role of Psychological Need Satisfaction in Relationships between Types of Passion for Sport and Athlete Burnout. J. Sports Sci..

[15] Paradis K.F., Cooke L.M., Martin L.J., Hall C.R. (2014). Just Need Some Satisfaction: Examining the Relationship between Passion for Exercise and the Basic Psychological Needs. Health Fit. J. Can..

[16] Parastatidou I.S., Doganis G., Theodorakis Y., Vlachopoulos S.P. (2012). Exercising with Passion: Initial Validation of the Passion Scale in Exercise. Meas. Phys. Educ. Exerc. Sci..

[17] Curran T., Hill A.P., Appleton P.R., Vallerand R.J., Standage M. (2015). The Psychology of Passion: A Meta-Analytical Review of a Decade of Research on Intrapersonal Outcomes. Motiv. Emot..

[18] Ryan R.M., Deci E.L. (2017). Self-Determination Theory: Basic Psychological Needs in Motivation, Development, and Wellness.

[19] Deci E.L., Ryan R.M. (2000). The “What” and “Why” of Goal Pursuits: Human Needs and the Self-Determination of Behavior. Psychol. Inq..

[20] de Charms R. (1986). Personal Causation: The Internal Affective Determinants of Behavior.

[21] White R.W. (1959). Motivation Reconsidered: The Concept of Competence. Psychol. Rev..

[22] Baumeister R.F., Leary M.R. (1995). The Need to Belong: Desire for Interpersonal Attachments as a Fundamental Human Motivation. Psychol. Bull..

[23] Ryan R.M., Deci E.L., Leary M.R., Tangney J.P. (2003). On Assimilating Identities to the Self: A Self-Determination Theory Perspective on Internalization and Integrity within Cultures. Handbook of Self and Identity.

[24] Lopes M., Vallerand R.J. (2020). The Role of Passion, Need Satisfaction, and Conflict in Athletes’ Perceptions of Burnout. Psychol. Sport Exerc..

[25] Verner-Filion J., Vallerand R.J., Amiot C.E., Mocanu I. (2017). The Two Roads from Passion to Sport Performance and Psychological Well-Being: The Mediating Role of Need Satisfaction, Deliberate Practice, and Achievement Goals. Psychol. Sport Exerc..

[26] Bartholomew K.J., Ntoumanis N., Ryan M.R., Thogersen-Ntoumani C. (2011). Psychological Need Thwarting in the Sport Context: Assessing the Darker Side of Athletic Experience. J. Sport Exerc. Psychol..

[27] Vansteenkiste M., Ryan R.M. (2013). On Psychological Growth and Vulnerability: Basic Psychological Need Satisfaction and Need Frustration as a Unifying Principle. J. Psychother. Integr.

[28] Uroh C.C., Adewunmi C.M. (2021). Psychological Impact of the COVID-19 Pandemic on Athletes. Front. Sports Act. Living.

[29] Jowett S., Shanmugam V., Schinke R., McGannon K.R., Smith B. (2016). Relational Coaching in Sport: Its Psychological Underpinnings and Practical Effectiveness. Routlegde International Handbook of Sport Psychology.

[30] Jowett S. (2017). Coaching Effectiveness: The Coach–Athlete Relationship at Its Heart. Curr. Opin. Psychol..

[31] Kenny D.A. (1996). Models of Non-Independence in Dyadic Research. J. Soc. Pers. Relat..

[32] Kenny D.A., Cook W.L. (1999). Partner Effects in Relationship Research: Conceptual Issues, Analytic Difficulties, and Illustrations. Pers. Relatsh..

[33] Kenny D.A., Kashy D.A., Cook W.L. (2006). Dyadic Data Analysis.

[34] #blijfsporten CODE GEEL—Update 14 Maart. https://www.sport.vlaanderen/media/16547/basisprotocol-sport.pdf.

[35] Coronavirus COVID-19. https://www.info-coronavirus.be/nl/news/.

[36] Chen B., Vansteenkiste M., Beyers W., Boone L., Deci E.L., van der Kaap-Deeder J., Duriez B., Lens W., Matos L., Mouratidis A. (2015). Basic Psychological Need Satisfaction, Need Frustration, and Need Strength across Four Cultures. Motiv. Emot..

[37] Delrue J., Soenens B., Morbée S., Vansteenkiste M., Haerens L. (2019). Do Athletes’ Responses to Coach Autonomy Support and Control Depend on the Situation and Athletes’ Personal Motivation?. Psychol. Sport Exerc..

[38] Reynders B., Vansteenkiste M., van Puyenbroeck S., Aelterman N., de Backer M., Delrue J., de Muynck G.-J., Fransen K., Haerens L., vande Broek G. (2019). Coaching the Coach: Intervention Effects on Need-Supportive Coaching Behavior and Athlete Motivation and Engagement. Psychol. Sport Exerc..

[39] Watson D., Clark L.A., Tellegem A. (1988). Development and Validation of Brief Measures of Positive and Negative Affect: The PANAS Scales. J. Pers. Soc. Psychol..

[40] Engelen U., de Peuter S., Victoir A., van Diest I., den Bergh O. (2006). Verdere Validering van de Positive and Negative Affect Schedule (PANAS) En Vergelijking van Twee Nederlandstalige Versies. Gedrag. Gezond..

[41] Rosseel Y. (2012). Lavaan: An R Package for Structural Equation Modeling. J. Stat. Softw..

[42] Vermote B., Waterschoot J., Morbée S., van der Kaap-Deeder J., Schrooyen C., Soenens B., Ryan R., Vansteenkiste M. (2022). Do Psychological Needs Play a Role in Times of Uncertainty? Associations with Well-Being During the COVID-19 Crisis. J. Happiness Stud..

[43] Lalande D., Vallerand R.J., Lafrenière M.K., Verner-Filion J., Laurent F., Forest J., Paquet Y. (2017). Obsessive Passion: A Compensatory Response to Unsatisfied Needs. J. Pers..

[44] Ackerman R.A., Kenny D.A. APIMPower: An Interactive Tool for Actor-Partner Interdependence Model Power Analysis [Computer Software] 2016. https://robert-a-ackerman.shinyapps.io/apimpower/.

